# Restricted Clonality and Limited Germinal Center Reentry Characterize Memory B Cell Reactivation by Boosting

**DOI:** 10.1016/j.cell.2019.11.032

**Published:** 2020-01-09

**Authors:** Luka Mesin, Ariën Schiepers, Jonatan Ersching, Alexandru Barbulescu, Cecília B. Cavazzoni, Alessandro Angelini, Takaharu Okada, Tomohiro Kurosaki, Gabriel D. Victora

**Affiliations:** 1 Laboratory of Lymphocyte Dynamics, The Rockefeller University, New York, NY, USA; 2 Weill Cornell/Rockefeller/Sloan Kettering Tri-Institutional MD-PhD Program, New York, NY, USA; 3 Universidade Federal do Rio de Janeiro, Rio de Janeiro, RJ, Brazil; 4 Department of Molecular Sciences and Nanosystems, Ca’ Foscari University of Venice, Venice, Italy; 5 European Centre for Living Technology (ECLT), Venice, Italy; 6 Laboratory for Tissue Dynamics, RIKEN Center for Integrative Medical Sciences, Yokohama, Kanagawa, Japan; 7 Graduate School of Medical Life Science, Yokohama City University, Yokohama, Kanagawa, Japan; 8 Laboratory of Lymphocyte Differentiation, WPI Immunology Frontier Research Center, Osaka University, Suita, Osaka, Japan; 9 Laboratory for Lymphocyte Differentiation, RIKEN Center for Integrative Medical Sciences, Yokohama, Kanagawa, Japan

**Keywords:** germinal center memory B cell, immunological memory, influenza A virus, clonal dynamics, antibody response, affinity maturation, memory reactivation

## Abstract

Repeated exposure to pathogens or their antigens triggers anamnestic antibody responses that are higher in magnitude and affinity than the primary response. These involve reengagement of memory B cell (MBC) clones, the diversity and specificity of which determine the breadth and effectiveness of the ensuing antibody response. Using prime-boost models in mice, we find that secondary responses are characterized by a clonality bottleneck that restricts the engagement of the large diversity of MBC clones generated by priming. Rediversification of mutated MBCs is infrequent within secondary germinal centers (GCs), which instead consist predominantly of B cells without prior GC experience or detectable clonal expansion. Few MBC clones, generally derived from higher-affinity germline precursors, account for the majority of secondary antibody responses, while most primary-derived clonal diversity is not reengaged detectably by boosting. Understanding how to counter this bottleneck may improve our ability to elicit antibodies to non-immunodominant epitopes by vaccination.

## Highlights

• Memory B cell reentry into germinal centers is rare under typical boost regimens
• Most (>90%) B cells in secondary GCs have no prior GC experience
• A clonality bottleneck restricts the diversity of recall antibody-producing cells
• Most primary diversity is found in an MBC compartment not accessed by boosting

## Graphical Abstract

**Figure:**
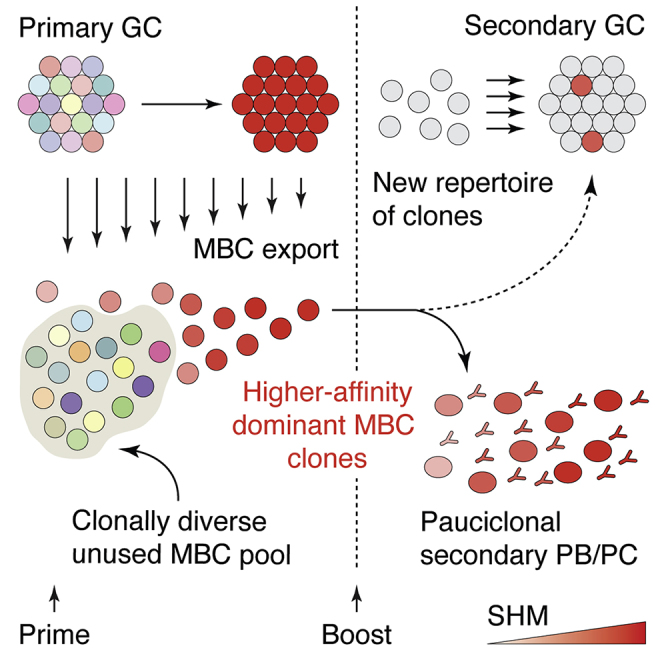

## Teaser

A clonal dynamics analysis of the transition between primary and recall B cell responses in mice reveals a clonality bottleneck that constricts the breadth of the secondary antibody response and limits reentry of previously matured B cells into secondary germinal centers.

## Introduction

One of the hallmarks of adaptive immunity is that the potency of immune responses increases, often dramatically, with repeated exposure totes an antigen. This is most evident in the humoral response, where antibodies generated the second time an antigen is encountered are both more abundant and of higher average affinity than those produced during the first encounter (Gray, 1993). This progressiveness is widely exploited by vaccination and underlies the need for booster doses to acquire and maintain the high serum antibody titers required for protection.

Enhanced secondary antibody responses are partly explained by the generation, by primary immunization, of a population of memory B cells (MBCs) (Gray, 1993, Kurosaki et al., 2015, Tarlinton and Good-Jacobson, 2013, Weisel and Shlomchik, 2017) that differentiate into antibody-secreting plasma cells (PCs) with extraordinary efficiency upon boosting (Askonas and Williamson, 1972, Moran et al., 2018, Weisel et al., 2010). At least a fraction of MBCs have undergone somatic hypermutation (SHM) and affinity maturation in germinal centers (GCs) formed during the primary response, which in general endows them with higher affinity for antigen when compared to their unmutated precursors (Berek et al., 1987, Siekevitz et al., 1987). This increased affinity synergizes with the efficiency with which MBCs differentiate into PCs to generate the high titers typical of the secondary response.

Whereas the formation and reactivation of MBCs have been studied in detail at the population level (Kurosaki et al., 2015, Weisel and Shlomchik, 2017), less is understood about how the clonal diversity of responding B cells, and ultimately of the PC compartment, is affected by the transition from primary to recall response. For example, our recent work shows that early GCs triggered by immunization with the model antigen chicken gamma globulin (CGG) contain on average 80–90 independently rearranged B cell clones per GC (Tas et al., 2016). Given that MBC generation in mice has been shown to occur most efficiently prior to or early during the GC reaction, when clonal diversity is at its peak (Weisel et al., 2016), the MBC repertoire is expected to be highly diverse. Notwithstanding, previous studies suggest that only a small number of B cell clones—usually mutated and affinity matured—are productively engaged by booster immunization, at least in the stereotypical (and *a priori* low diversity) responses of mice to haptens (Blier and Bothwell, 1987, Liu et al., 1996). Clarifying these dynamics may help explain immunological phenomena such as immunodominance and “original antigenic sin” (Fazekas de St. Groth and Webster, 1966a, Fazekas de St. Groth and Webster, 1966b) and can contribute to our understanding of the rules governing the response to immunization in the presence of previous immunity to an antigen, as is almost always the case with influenza (Victora and Wilson, 2015).

In addition to rapidly differentiating into PCs, at least some populations of MBCs have the ability to reenter GC reactions upon recall immunization. The rules controlling GC reentry are currently a topic of interest (Dogan et al., 2009, McHeyzer-Williams et al., 2015, McHeyzer-Williams et al., 2018, Pape and Jenkins, 2018, Pape et al., 2011, Shlomchik, 2018, Zuccarino-Catania et al., 2014). Most studies agree that a subset of MBCs defined either by carrying an immunoglobulin M (IgM) B cell receptor (Dogan et al., 2009, Pape et al., 2007) or by the absence of markers of more mature memory (Zuccarino-Catania et al., 2014) have the potential to reenter GCs when adoptively transferred into different types of recipient mice. However, with one exception (McHeyzer-Williams et al., 2015), these studies do not address whether this potential is realized under non-transfer conditions, where numbers of memory B and T cells as well as preexisting antibody titers could all play a role. Critically, none of these studies address the relative contribution to secondary GCs of naive-derived B cells, which could potentially compete with MBC-derived clones, restricting their ability to rediversify in secondary responses. Resolving this issue will be important for any attempts to elicit the expansion and hypermutation of B cell clones with defined specificities by iteratively recalling MBCs to sequential GC reactions, as is thought to be required for the generation of broadly neutralizing antibodies to influenza and HIV by vaccination (Burton et al., 2012).

To clarify these points, we carried out a clonal analysis of the response to protein boosting in mice primed either by protein immunization or by influenza virus infection. We show that, contrary to our expectations, recall GCs are composed overwhelmingly of clones without prior GC experience, likely naive in origin, and rediversification of previously matured MBCs in secondary GCs is rare and restricted to a small contingent of clones. Although a larger fraction of secondary PCs and plasmablasts (PBs) is MBC derived, these compartments are also limited to few clones, while most primary-derived diversity can be found within a pool of largely IgM^+^ MBCs that is not productively engaged by boosting. These findings identify hurdles that may need to be overcome when attempting to elicit highly mutated antibodies to non-immunodominant epitopes, as is thought to be required for effective vaccination against influenza and HIV.

## Results

**Figure 1:**
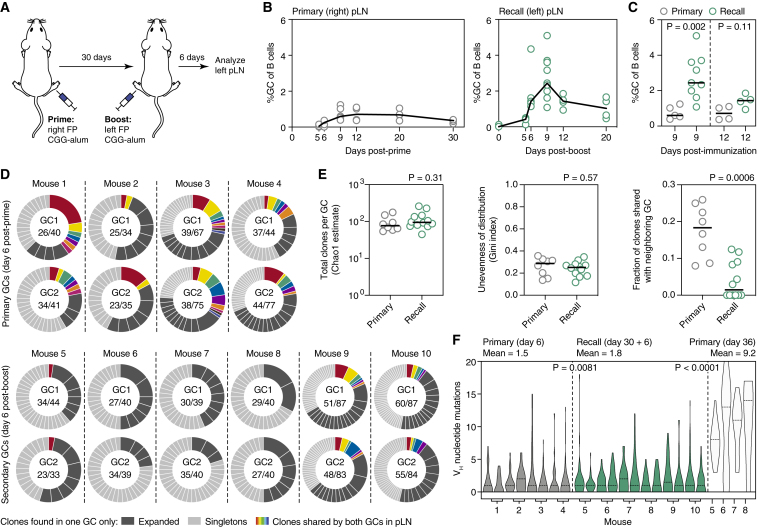
Secondary GCs Are Clonally Diverse and Have Low SHM Load (A) Schematic representation of the immunization protocol. (B and C) Kinetics of primary and recall GC responses in wild-type mice immunized and boosted (B) as in (A), summarized in (C). Graphs indicate the percentage of GC B cells (CD38^low^Fas^hi^) among total B cells. Each symbol represents one mouse; lines represent median. (D) Clonal diversity of individual early primary GCs (reanalyzed from Tas et al., 2016) or early recall GCs (this study) obtained by *in situ* photoactivation. PA-GFP transgenic mice were crossed to *Rosa26*^Lox-Stop-Lox-tdTomato^ and *Aicda*^Cre/+^ (primary) or *Ighg1*^Cre/+^ (recall) for visualization of GCs prior to photoactivation. Numbers are (clones observed)/(cells sequenced). Two GCs were sequenced from each pLN; colored slices represent clones that were found in both GCs from the same node. (E) Clonal richness (Chao1 estimator, downsampled to the smallest no. of cells, Left), evenness of clonal distribution (Gini index, Center), and sharing between neighboring GCs (Right) in early primary and early recall GCs. Each symbol represents one GC, with two GCs per mouse as in (D); lines represent median. (F) Distribution of somatic mutations per B cell in early primary and early recall GCs. Each bar/violin plot represents one GC, with two GCs per mouse as in (D). Dashed lines represent median. All data are pooled from at least two independent experiments. p values are for Mann-Whitney U test (C and E) and Kruskal-Wallis test with Dunn’s multiple comparison test (F).

To investigate the clonal dynamics of the recall B cell response, we first immunized mice subcutaneously (s.c.) in the right hind footpad (FP) with the model antigen CGG in alum adjuvant to generate a primary GC in the draining popliteal lymph node (pLN). Thirty days later, when primary GCs have largely subsided (Figure 1B), we boosted the contralateral FP of the same mouse with the same protein and adjuvant combination to generate a recall response (Figure 1A). This anatomical segregation ensures that the recall response is generated *de novo* from circulating MBCs, rather than by reactivation of B cells still present in residual GCs in the primary lymph node (LN). GCs in the recall (left) pLN are readily detectable at 6 days and reach peak size at 9 days post-boost (Figure 1B). As classically described for the secondary response (Liu et al., 1991), boost-derived GCs reached higher peak size and decayed more rapidly than those formed by primary immunization (Figure 1C), confirming the anamnestic nature of the response.

### Secondary GCs Are Clonally Diverse and Have Low SHM Load

We began by determining the clonal composition of recall GCs in this model. We hypothesized that, if recall GCs rely primarily on MBCs generated from the most expanded and affinity-matured clones of the primary response, they would be enriched in larger clones, with higher SHM load, and that would be present across multiple GCs in the boosted pLN. Using *in situ* photoactivation to isolate B cells from individual GCs by flow cytometry (Tas et al., 2016, Victora et al., 2010), we sequenced the *Ig* genes (Tiller et al., 2009) of single B cells from two GCs per pLN obtained at 6 days after boost (Figure 1D). For reference, we compared these data to our previously published analysis of day 6 primary GCs under similar conditions (Tas et al., 2016).

Counter to our prediction, secondary GCs were at least as clonally diverse as primary GCs (mean Chao1 estimated richness = 94 versus 118 clones per GC, Gini index (a measure of the unevenness of expansion across clones) = 0.26 versus 0.24 for primary and recall GCs, respectively) (Figures 1D and 1E). The fraction of clones present in both GCs from the same pLN was lower in the recall than in the primary response (mean 17% versus 4.1% of clones also found in the neighboring GC in primary and recall responses, respectively), suggesting that most of the precursors of secondary GC B cells were not present in multiple copies prior to boosting (Figures 1D and 1E). B cells in recall GCs had SHM loads (Figure 1F) that were much lower than those of B cells sorted from the residual primary GCs still present in the right pLN at day 36 post-priming (mean V_H_ nucleotide SHM = 1.8 versus 9.2, respectively) and only slightly higher than those of early primary GC B cells (mean = 1.5), possibly because our earlier data were obtained from mice with only one intact copy of activation-induced deaminase (AID) (Tas et al., 2016). Thus, the clonal diversity and SHM load of early recall GCs resemble those of early primary GCs, rather than those of more recently generated memory. Rediversification of heavily mutated and expanded MBC clones is therefore not a prominent feature of the secondary GC response in this setting.

### Secondary GCs Contain Mostly B Cells without Primary GC Experience

**Figure 2:**
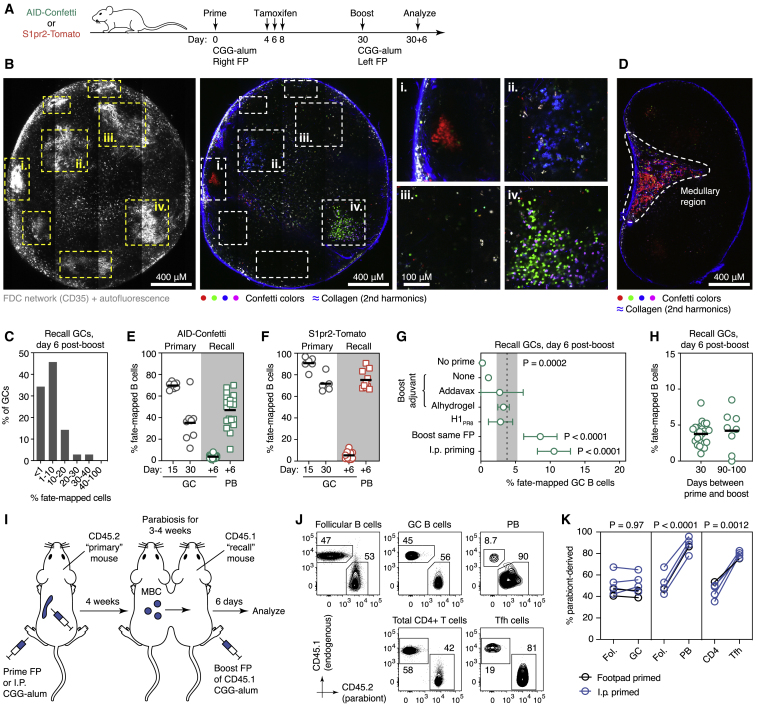
Secondary GCs Are Composed Primarily of B Cells without Prior GC Experience (A) Experimental protocol for (B)–(F). (B) Multiphoton image showing GCs in the left pLN at 6 days post-boost. GCs (dashed lines) were identified by presence of follicular dendritic cell (FDC) networks (labeled *in vivo* using a far-red anti-CD35 antibody) and autofluorescent tingible body macrophages (leftmost panel; image is a collapsed 40 μm, 3-slice *z* stack). Confetti colors and collagen fibers (second harmonics, blue) for a single Z slice are shown in the central panel. GCs marked with roman numerals are magnified in the smaller panels to the right. (C) Distribution of GCs from five pLNs according to fate-mapped cell density, quantified from images as shown in (B). (D) Image of explanted lymph node slice showing accumulation of fluorescent cells in medullary region. (E and F) Percentage of fate-mapped cells in the indicated compartments by flow cytometry, in AID-Confetti (E) or S1pr2-Tomato (F) mice. Each symbol represents one mouse; lines represent mean. Recall GCs and PBs are from the same sample. All data are from at least 2 independent experiments. (G) Percentage of fate-mapped cells in recall GCs in AID-Confetti mice immunized using different protocols. Dashed line and shaded area represent mean ± SD of standard protocol (day +6 GC from E). Rows are mean ± SD for 3–9 mice from at least two independent experiments per condition. p values are for one-way ANOVA with Dunnett’s multiple comparison test; only p < 0.05 is shown. (H) Percentage of fate-mapped cells in AID-Confetti mice boosted 30 or 90–100 days after priming. Each symbol represents one mouse; bars represent median. Data for day 30+6 are the same as in (E). (I) Experimental protocol for (J) and (K). (J) Flow cytometry of the boosted pLN of the recall mouse showing fraction of cells originating from primary (CD45.2) and recall (CD45.1) parabionts. Primary mouse in this example was immunized i.p. (K) Quantification of data from multiple pairs from 3–4 independent experiments, primed i.p. (blue) or s.c. (black). Each symbol represents one parabiont pair. p values are for paired Student’s t test.

**Figure S1:**
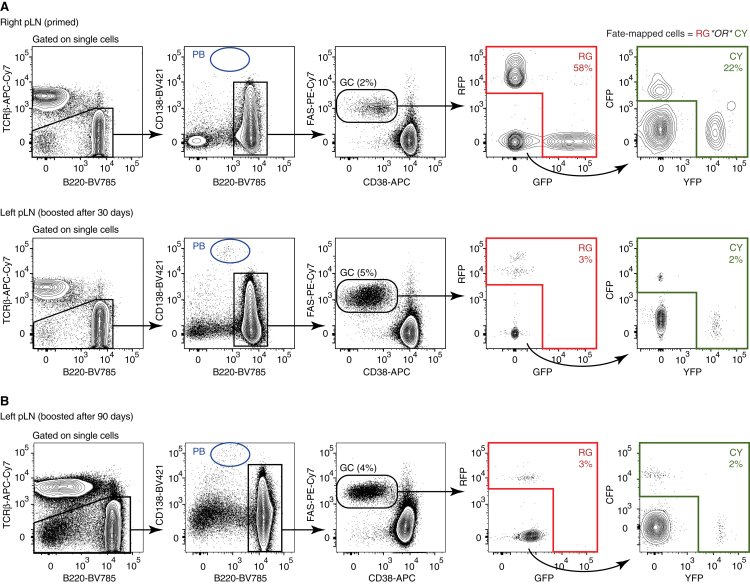
Flow Cytometry Gating Strategy for Identifying Fate-Mapped GC B Cells in the AID-Confetti Model, Related to Figure 2 (A) Mice were primed and boosted as in Figure 2A. Flow cytometry plots show cells from the primed right pLN and the boosted left pLN. The final fate-mapped gate was a Boolean “OR” gate combining fluorescence in the four Confetti colors. A similar strategy was used to identify fate-mapped plasmablasts, starting from the “PB” gate shown in blue. (B) Flow cytometry showing fate-mapping in secondary GCs for a mouse primed as in (A) but boosted after 90-100 days.

Our photoactivation experiments showed that recall GCs consisted primarily of B cells with few if any signs of previous GC experience. To test this formally, we fate-mapped B cells activated during the primary response using the *Aicda*^CreERT2/+^.*Rosa26*^Confetti/Confetti^ (AID-Confetti) mouse model, in which recombination of a “Brainbow” multicolor fate-mapping allele is induced in B cells expressing AID (encoded by *Aicda*) upon administration of tamoxifen (Dogan et al., 2009, Livet et al., 2007, Snippert et al., 2010, Tas et al., 2016) and then followed the fate of labeled cells after boosting (Figure 2A). Treatment with tamoxifen by gavage at days 4, 6, and 8 after primary immunization (which spans the late-pre-GC and early GC stages; Schwickert et al., 2011) led to recombination of ∼70% of all GC B cells, as measured 7 days after the final tamoxifen dose. Imaging of explanted pLNs at 6 days post-boost revealed that fate-mapped B cells were relatively rare in recall GCs. One-third of these structures (34%) were entirely devoid of fate-mapped B cells, and another 46% had <10% labeled B cells (Figures 2B and 2C). By contrast, fate-mapped cells were abundant in the LN medullary region, where LN-resident PBs are expected to accumulate (Figure 2D). Quantification of fate-mapped cells by flow cytometry (as detailed in Figure S1) confirmed these observations: whereas on average 70% of primary GC B cells were labeled at day 15 after immunization and 35% of cells were still labeled in residual GCs at day 30, only 3.8% of B cells in recall GCs were labeled at 6 days after boost (Figure 2E). As suggested by the concentration of fluorescent cells in the LN medulla, an average of 47% of local PBs were fate-mapped when assayed by flow cytometry (Figure 2E). Thus, the scarcity of fate-mapped clones in recall GCs was not due to a failure to generate recallable fate-mapped MBCs. Identical results were obtained with a different fate-mapping system where the GC-specific and highly efficient *S1pr2*-CreERT2 bacterial artificial chromosome (BAC) transgenic strain (Shinnakasu et al., 2016) was used to drive recombination of a *Rosa26*^Lox-Stop-Lox-tdTomato^ cassette (S1pr2-Tomato mice) (Figure 2F).

**Figure S2:**
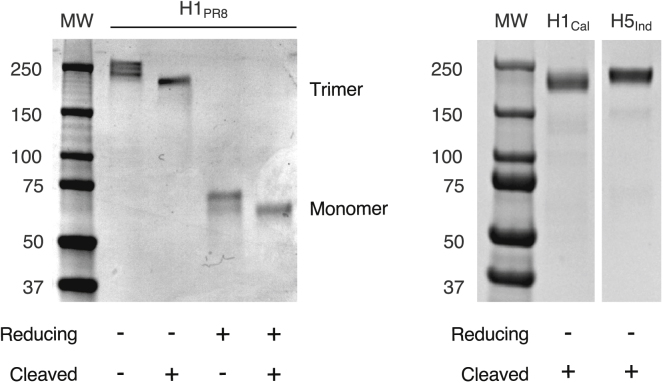
Production of Recombinant HAs, Related to Figure 2 Cysteine-stabilized HAs (Lee et al., 2015) were produced in CHO cells and purified as detailed in the STAR Methods. *Left*, stained SDS-PAGE gel of H1_PR8_ under reducing and non-reducing conditions. HA is shown prior to and after thrombin digestion to remove trimerization, biotinylation, AviTag, and HisTag domains. *Right*, non-reducing SDS-PAGE gel of HAs used for heterologous boosting (pandemic H1 A/California/07/2009 (H1_Cal_), and H5 A/Indonesia/05/2005 (H5_Ind_)) after thrombin digestion. Both strips are cropped from the same gel.

We performed a series of controls to rule out that inefficient recruitment of GC-experienced MBCs to recall GCs was due to our choice of prime-boost protocol (Figure 2G). These included: omitting the primary immunization (while maintaining the tamoxifen treatment) to ascertain that labeled cells in the recall were indeed primary derived; omitting the adjuvant from the boost; changing both prime and boost adjuvants to a squalene-based formulation (Addavax) or to a more potent form of alum (alhydrogel) (Cain et al., 2013); and changing immunogen from CGG to a recombinant cysteine-stabilized trimeric form of influenza hemagglutinin (HA) H1 A/Puerto Rico/08/1934 (H1_PR8_) (Figure S2), none of which substantially increased the participation of fate-mapped cells in secondary GCs. Boosting the same LN used for primary immunization resulted in GCs that contained twice the frequency of fate-mapped cells as contralateral boosting (8.6% versus 3.8%), possibly because any recruitment of naive or MBCs in this node is superimposed on a refueling of the ongoing GC reaction by the boost (Schwickert et al., 2009, Shulman et al., 2013). Priming by intraperitoneal (i.p.) injection increased participation of fate-mapped cells in recall GCs to 11%, suggesting that increasing the number of GC-experienced MBCs generated in the primary response can have a marginal effect on their participation in recall GCs. However, because i.p. priming often induces GCs also in pLNs (unpublished data), a contribution from an ongoing GC reaction in the pLN prior to boosting cannot be ruled out. Thus, predominance of non-fate-mapped cells in secondary GCs is robust to common variations in the prime-boost protocol. Previous work in humans has shown that MBCs may be refractory to reentering GCs for a period of time after their generation (Lau et al., 2017). However, delaying boosting from 1 to 3 months after priming yielded secondary GCs with similarly low proportions of fate-mapped B cells (Figures 2H and S1B), indicating that such a restriction cannot account for our findings.

The unlabeled majority of B cells in recall GCs could derive either from naive B cells or from early MBCs generated prior to GC formation, which may never have expressed *Aicda* or *S1pr2* while proliferating (Kaji et al., 2012, Weisel et al., 2016). As an attempt to distinguish between these possibilities, we immunized wild-type (WT) mice (the “primary” mouse) either i.p. or in one FP with CGG in alum and, 4 weeks later, joined these mice parabiotically to an allelically marked unimmunized partner (the “recall” mouse, Figure 2I). Because the recall mouse was never in contact with antigen prior to parabiosis, all CGG-specific MBCs, including any early memory, must have derived from the primary mouse. Boosting the recall mouse invariably generated GCs that contained a proportion of primary mouse-derived cells close to that found in the naive B cell compartment of the same pLN, regardless of site of priming (Figures 2J and 2K). In contrast, secondary PBs were strongly skewed toward the primary mouse, indicating that detectable B cell memory was generated and confirming that secondary PBs are mostly MBC derived (Figures 2J and 2K). Of note, T follicular helper (Tfh) cells were also skewed toward the primary mouse (Figures 2J and 2K); therefore, inefficient participation in recall GCs applies only to MBCs and not to memory T cells, as classically predicted by prime-boost experiments (Liu et al., 1991). Together, our data show that secondary GCs, while allowing reentry of a small contingent of MBCs, are seeded primarily by B cells without prior GC experience or strong evidence of clonal expansion, which supports a model in which they arise directly from naive precursors.

**Figure 3:**
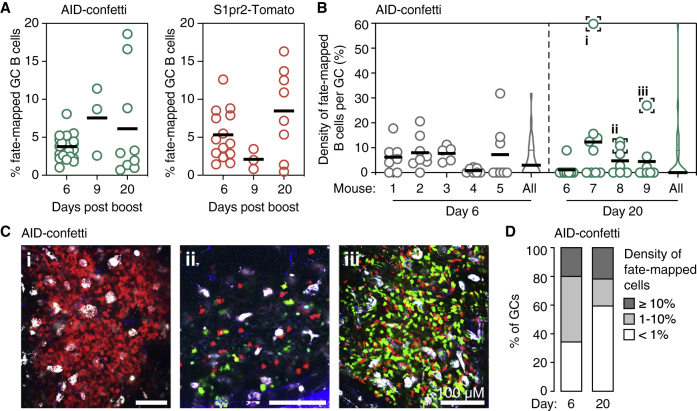
B Cells with Primary GC Experience Are Not at a Competitive Advantage in Secondary GCs Experimental design as in Figure 2A. (A) Change over time in the percentage of fate-mapped cells in secondary GCs by flow cytometry, in AID-Confetti (Left) or S1pr2-Tomato mice (Right). Each symbol represents one mouse; lines represent mean. All data are from at least 2 independent experiments. Data for day 6 are reproduced from Figures 2E and 2F. (B) Quantification of the fraction of fate-mapped B cells in individual GCs by two-photon microscopy at 6 and 20 days post-boost. Each symbol represents one GC and violin plots show aggregate data from all mice; lines represent mean. Data are from 2–3 independent experiments with at least six GCs analyzed per LN. (C) Two-photon images of GCs containing fate-mapped B cells (roman numerals correspond to those indicated in B). (D) Percentage of GCs with high (>10%), mid (1%–10%), and no/low (<1%) fate-mapped B cell content. Note the increase in GCs with <1% labeled cells at the late time point. Data are the same as in (B).

Following the abundance of fate-mapped B cells in recall GCs (generated as in Figure 2A) by flow cytometry over time showed no discernible increase in the overall proportion of fate-mapped cells, neither in the short term (day 9) nor in the longer term (day 20) (Figure 3A). While memory-derived clones occasionally showed signs of burst-like positive selection—the largest MBC expansion reaching an estimated 60% of the total GC population (Figures 3B and 3C)—these clones were just as likely to be eliminated from recall GCs, accounting for the unchanging average value (Figure 3D). Therefore, not only are cells with prior GC experience a minor component of secondary GCs, but prior GC experience is also not sufficient to ensure that these clones have a selective advantage when competing against naive-like clones.

We conclude that secondary GCs are primarily sites of *de novo* affinity maturation of unmutated B cell clones, rather than of rediversification of previously matured MBCs.

### The Memory-Derived B Cell Response Is Clonally Restricted

**Figure 4:**
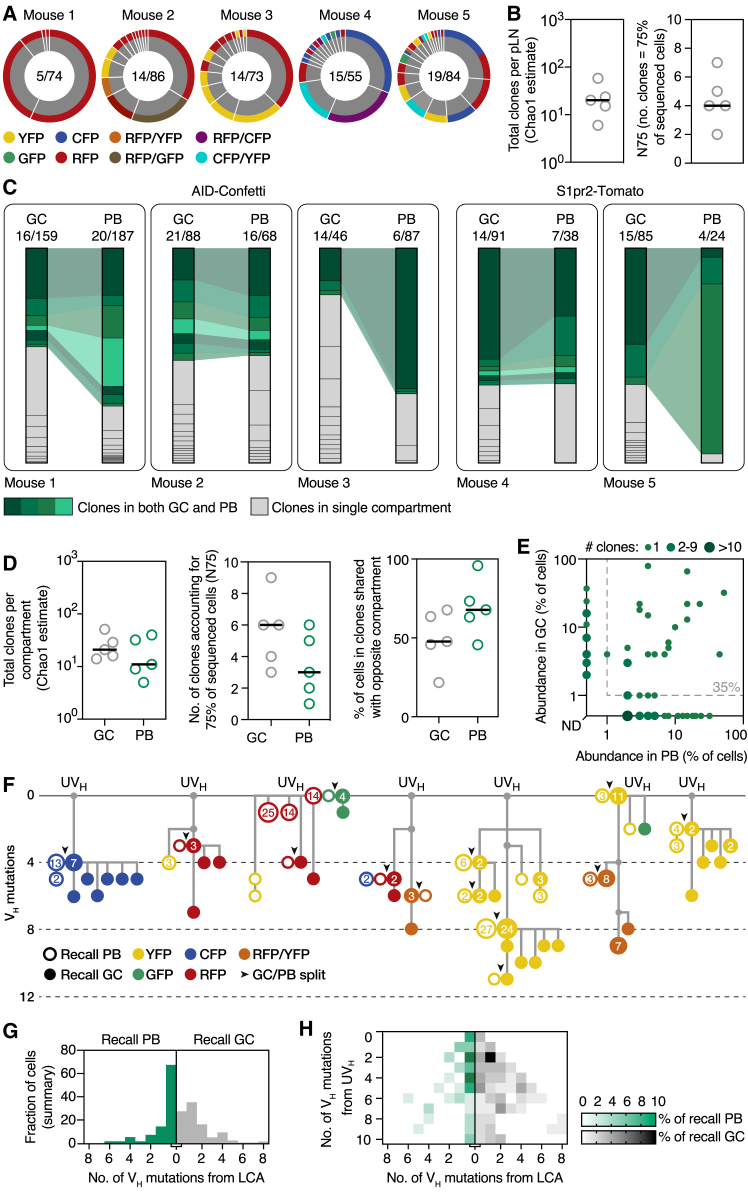
Clonal Dynamics of Secondary GC and PB Responses Experimental design as in Figure 2A. (A) Pie charts showing clonal distribution of all fate-mapped GC B cells from individual boosted pLNs. Each slice in inner (gray) rings represents one clone (distinct V(D)J rearrangement); outer rings show Confetti color. Numbers are (clones observed)/(cells sequenced). (B) Quantification of data in (A). Each symbol represents one pLN; bar represents median. (C) Clonality maps showing distribution and sharing of clones between secondary GC and PB compartments from the same pLN in AID-Confetti and S1pr2-Tomato mice. Each slice of a column represents an individual clone; each column represents one LN. GC/PB pairs are from the same pLN. Clones found in both compartments are connected and colored green. Numbers are as in (A). (D) Quantification of data in (C). Each symbol represents GCs or PBs from one pLN; bar represents median. (E) Scatterplot showing abundance of expanded clones (>1 copy) in PB and GC compartments in the same pLN. Data are from (C). Each symbol represents one clone. Presence of >1 clone in same X-Y position is denoted by larger/darker symbols. 35% of expanded clones had both GC and PB members. (F) Trees showing phylogenetic relationships between V_H_ sequences (excluding CDR3) of cells from selected clones. The top line represents the clone’s unmutated V_H_ region (UV_H_). Numbers inside cells indicate how many times a particular sequence/color/cell-type combination was observed. PB and GC B cells sharing the same V_H_ sequence are shown as adjacent circles and marked by black arrowheads. Symbols colored according to Confetti colors. (G and H) Distance between observed PB or GC B cell sequences and their last common ancestor (LCA) with a cell in the opposite compartment, shown as a histogram (G) or as a heatmap according to distance from UV_H_ (H). Note that most PBs are identical (0 mutations distant) from their LCA with a GC B cell, indicative of a single MBC of origin. Data are from (C).

To investigate the clonal dynamics underlying the scarcity of GC-experienced B cells in recall GCs, we sequenced the *Ig* genes of fate-mapped B cells sorted from GCs of AID-Confetti or S1pr2-Tomato mice at 6 days after boosting. As suggested by the low color diversity of recall GCs in the Confetti model (Figure 2B), fate-mapped GC B cells were focused on a small number of clones (Figure 4A). A median of 20 total clones (Chao1 estimate) was present in the entire boosted LN, and 4 clones were sufficient to account for 75% of fate-mapped cells sequenced (*N75* index; see STAR Methods) (Figure 4B), in marked contrast to the hundreds of clones found in primary GCs at the corresponding time point (Tas et al., 2016 and Figures 1D and 1E).

A bias of MBCs toward PB and PC fates has been identified in different models (Arpin et al., 1997, Dogan et al., 2009, Kometani et al., 2013, Pape et al., 2011, Zuccarino-Catania et al., 2014) and could explain the higher abundance of MBC-derived cells in the PB compartment in our experiments (Figures 2D–2F). To investigate this possibility, we sequenced the *Ig* genes of fate-mapped secondary PB and GC B cells from the same boosted pLNs. Despite the higher proportion of fate-mapped PBs, GC and PB compartments were both low in diversity, with a median 21 and 11 total clones per LN (Chao1) and an N75 of only 6 and 3 clones per LN in GC and PB compartments, respectively (Figure 4D). Furthermore, the same clone was often found as both a GC B cell and a PB (∼50% of GC B cells were part of clones found also among PBs and ∼70% of PBs belonged to clones also found in the GC [Figures 4C and 4D], and 35% of all expanded clones [>1 cell] were found in both compartments [Figure 4E]). SHM analysis showed evidence of repeated recruitment of MBCs from within each clone (recalled PBs mapping to multiple points within the same clonal tree in Figure 4F). This was confirmed by the presence of cells expressing different Confetti color combinations within the same phylogeny, which, given that Cre recombination occurred during the primary response, must originate from different MBCs (Figure 4F). Moreover, single MBCs often generated both GC and PB progeny (arrowheads in Figure 4F), as evidenced by the observation that, within clones that contained both cell types, 67% of recalled PBs were identical in V_H_ sequence to their last common ancestor with a recalled GC B cell (Figures 4G and 4H). Thus, diversion of MBCs toward the PB fate cannot account for the scarcity of MBC-derived B cells in secondary GCs in this model.

We conclude that, given that the entire MBC-derived secondary response is clonally restricted, the low frequency and diversity of MBC-derived secondary GC B cells cannot be explained by diversion of MBCs toward the PB fate.

### MBCs Harbor a Reservoir of Clonal Diversity Not Detectably Engaged by Boosting

The contrast between the hundreds of B cell clones recruited to a primary response (Tas et al., 2016) and the oligoclonality of the MBC-derived secondary response suggests that a large fraction of primary clonal diversity is not productively engaged by boosting. To probe for this missing diversity, we used a second experimental system where mice are primed by infection with mouse-adapted influenza (strain PR8) and then boosted subcutaneously with recombinant H1_PR8_ protein in adjuvant. In addition to being clinically relevant—humans pre-exposed to influenza by infection are commonly vaccinated intramuscularly with inactivated virus or protein preparations—influenza infection in mice produces a population of MBCs that can be reliably identified using HA tetramers (Frank et al., 2015, Whittle et al., 2014), allowing us to measure the full diversity of MBC clones at the same time as we trace their boost-expanded progeny.

**Figure 5:**
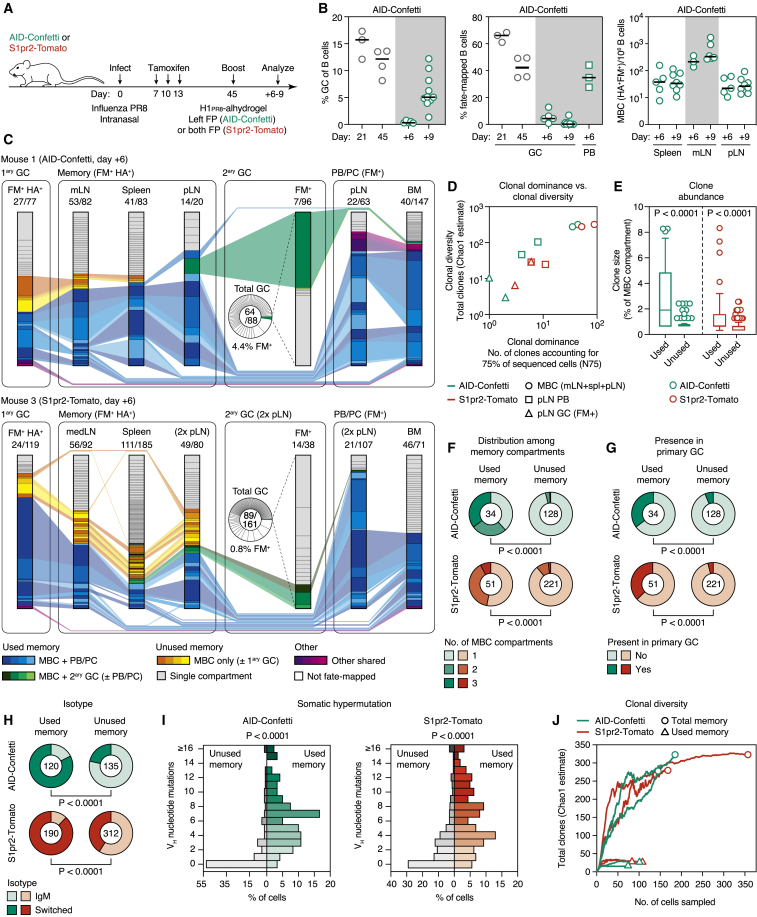
Clonal Dynamics of the Response to HA Immunization following Influenza Infection (A) Experimental protocol for Figures 5B–5J. (B) (Left) GC size (percentage of GCs of all B cells); (Center) percentage of fate-mapped cells in GC and PB compartments; and (Right) MBCs per 10^6^ B cells, at different time points after primary influenza PR8 infection (gray symbols) or boosting with homologous H1_PR8_ protein (green symbols). Each symbol represents one mouse; bar represents median; data are pooled from 2–3 independent experiments. Equivalent data for the S1pr2-Tomato strain are presented in Figure S3B. (C) Clonality maps tracking fate-mapped clones across multiple compartments. FM^+^, only fate-mapped cells sorted; HA^+^, only H1_PR8_-binding cells sorted. Clones present in more than one compartment are connected and depicted in color. Pie chart insets show clonal distribution in the entire secondary GC, including non-fate-mapped cells. Equivalent data for 2 additional mice is shown in Figure S4A; clonal diversity and dominance data for all mice are summarized in Figure S4B. (D–J) Analysis of data depicted in (C) and Figure S4A. (D) Estimated clonal diversity (Chao1) and dominance (N75) of the indicated compartments. Each symbol represents one mouse. (E–J) Characteristics of MBC clones responding (used) and not detectably responding (unused) to secondary immunization. (E) Memory clone size, given as a percentage of the total memory compartment of each mouse. Each symbol represents one clone, and boxplots are median and quartiles; whiskers are 10^th^ and 90^th^ percentiles. Cells with the exact same sequence are collapsed into one data point. (F) Number of memory compartments spanned by each MBC clone. (G) Fraction of all memory clones still present in the primary (mLN) GC. For (F) and (G), number of clones analyzed is indicated in each chart. (H) Percentage of IgM^+^ cells among used and unused MBC clones. Number of cells analyzed is indicated in each chart. (I) Distribution of somatic mutations among used and unused MBC clones. Data are collapsed by sequence as in (E). (J) Estimated total number of distinct clones (Chao1) among all MBCs (circles) and used MBCs (triangles), with downsampling analysis. Each line/symbol represents one mouse. For (E)–(I), sequences are pooled from 2 mice per genotype. p values are for Mann-Whitney U test (E and I) and chi-square test (F–H).

**Figure S3:**
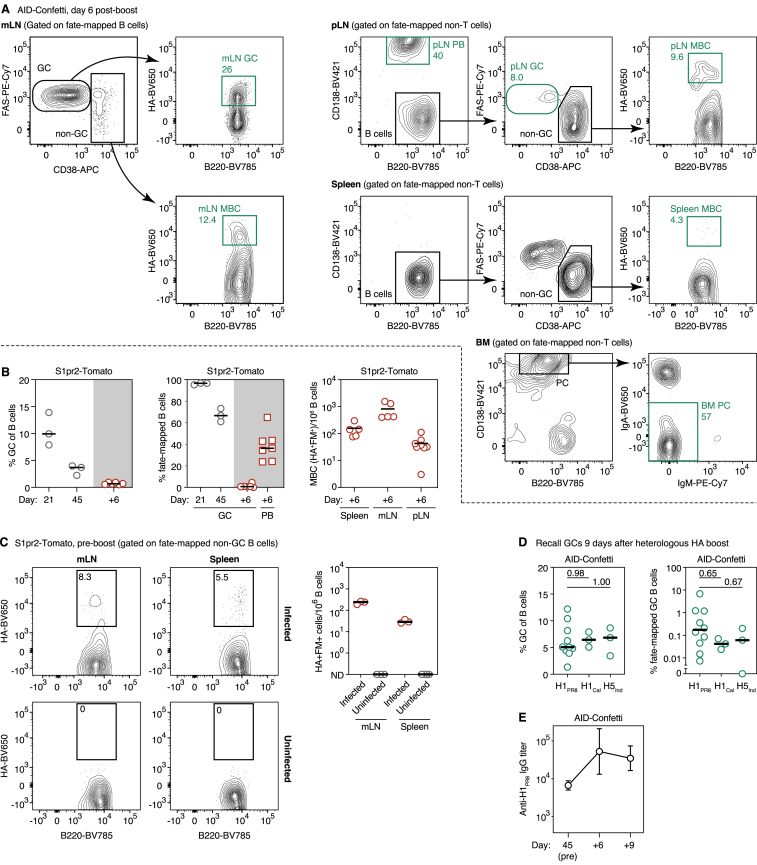
Flow Cytometric Analyis of Fate-Mapped B Cell Responses Following Influenza Infection, Related to Figure 5 (A) Gating strategy used for sorting the cell populations sequenced in Figures 5C and S4A. Only AID-Confetti mice are shown. Mice were infected and boosted as in Figure 5A. Plots are from day 6 post-boost. Gating is shown for mLN, boosted pLN, spleen, and BM. Gates sorted for sequencing are in green (percentage of parent indicated), and correspond to the cell populations shown in the clonality map in Figure 5C. (B) Fate-mapping of primary and recall cells in S1pr2-Tomato, as shown for AID-Confetti in Figure 5B. *Left*, GC size (% GC of all B cells); *center*, % fate-mapped cells in GC and PB compartments; and *right*, MBC per 10^6^ B cells, at different time points after primary influenza PR8 infection (gray symbols) or boosting with homologous H1_PR8_ protein (red symbols). Each symbol represents one mouse, bar represents median; pooled from 1-3 independent experiments. (C) Absence of fate-mapped HA-binding B cells in mLN and spleen of uninfected S1pr2-Tomato mice. Uninfected mice were treated as in Figure 5A, but infection was omitted. Plots show the pre-boost time point. Graph shows quantification for three mice per condition from different experiments. ND, none detected. (D) Proportion of fate-mapped cells in secondary GCs generated as in Figure 5A but boosted with heterologous HA strains (H1_Cal_, pandemic H1 A/California/07/2009; H5_Ind_, H5 A/Indonesia/05/2005) and assayed at 9 days post-boost. Data for HA_PR8_ are reproduced from Figure 5B for comparison. Bars represent medians. P values are for one-way ANOVA with Dunnett’s multiple comparison test. (E) Increase in serum antibody titers to H1_PR8_ upon protein boosting. Mice infected and boosted as in Figure 5A. The day 45 sample is pre-boost. Geometric mean + SD for 3-10 mice from at least 2 independent experiments are shown.

We primed AID-Confetti or S1pr2-Tomato mice by intranasal infection and fate-mapped activated B cells by tamoxifen administration at 7, 10, and 13 days post-infection, covering the initial period of GC formation. On day 45, we boosted mice in one (AID-Confetti) or both (S1pr2-Tomato) FPs with recombinant H1_PR8_ in alhydrogel (Figure 5A). Infection elicited a strong GC response in the mediastinal (m)LN, which was still ongoing and efficiently fate-mapped at 45 days after infection (Figures 5B, S3A, and S3B). As expected, we were able to identify a clear population of MBCs (defined as CD38^+^ B cells that were fate-mapped and bound the HA tetramer) in the mLN, spleen, and boosted pLN at this time point (Figures 5B, S3A, and S3B). This population was completely absent from uninfected tamoxifen-treated controls, confirming the specificity of tetramer staining in the fate-mapped population (Figure S3C). As with our CGG prime-boost model (Figure 2E), the fraction of fate-mapped cells in recall GCs was low, decreasing from a median of 4.4% in incipient GCs at day 6 to 0.18% in the larger GCs found at day 9 post-boost (Figure 5B). Boosting with variant recombinant HAs (pandemic H1 A/California/07/2009 and H5 A/Indonesia/05/2005) yielded similar results (Figure S3D). Thus, the generation of a large MBC compartment by influenza infection was not sufficient to overcome the dominance of non-fate-mapped B cells in secondary GCs, regardless of whether boosting was homologous or heterologous.

**Figure S4:**
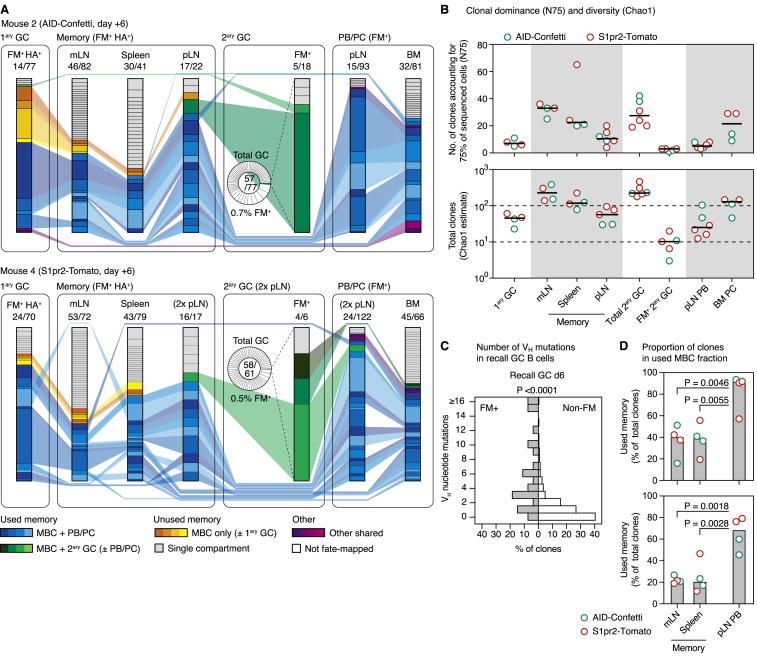
Clonal Analysis of the Response to HA Immunization Following Influenza Infection, Related to Figure 5 (A) Clonal maps showing distribution of clones across different compartments 6 days post-boost. Data as in Figure 5C, showing two additional mice. In mouse 4, FM+ cells were present in only one pLN GC. (B) Quantification of clonal dominance (N75) and total diversity (Chao1) in different compartments for all 4 mice. Data are for two AID-Confetti and two S1pr2-Tomato mice (Figures 5C and S4A). Each symbol represents one mouse, except for S1pr2-Tomato pLN cells, where both FPs were boosted and each symbol represents one pLN. One sample of FM^+^ pLN GC B cells from S1pr2-Tomato was omitted from the analysis due to low cell count. Bar represents median. (C) Average V_H_ mutations per clone in fate-mapped and non-fate-mapped GC B cells from Figures 5C and S4A (all four mice are pooled). P value is for Mann-Whittney U test. (D) Proportion of cells (left) and clones (right) found in the “used” MBC fraction (blue and green colors in Figures 5C and S4A). P value is for one-way ANOVA with Dunnett’s multiple comparisons test.

To probe for the missing clonal diversity, we sequenced *Ig* genes from single-sorted B cells from multiple compartments (GCs, MBCs, and PBs/PCs from the primary mLN, recall pLN, spleen, and bone marrow [BM]) at 6 days after homologous boosting, a time point when all of these populations were consistently present (Figure S3A). For primary GCs and all MBCs, we sorted only H1_PR8_-binding (HA^+^) fate-mapped (FM^+^) B cells, whereas we sorted all FM^+^ cells from recall GC and PB compartments (BM PCs were additionally restricted to IgM^–^IgA^–^ cells to decrease contamination by bystander (e.g., gut derived) GC responses). We also sorted non-fate-mapped B cells from the recall GC (pie charts in Figure 5C) to determine the full diversity of this compartment. The clonal maps in Figures 5C and S4A show the clonal distribution of cells in each compartment and the clonal relationships between compartments. Diversity data are summarized in Figures 5D and S4B.

**Figure S5:**
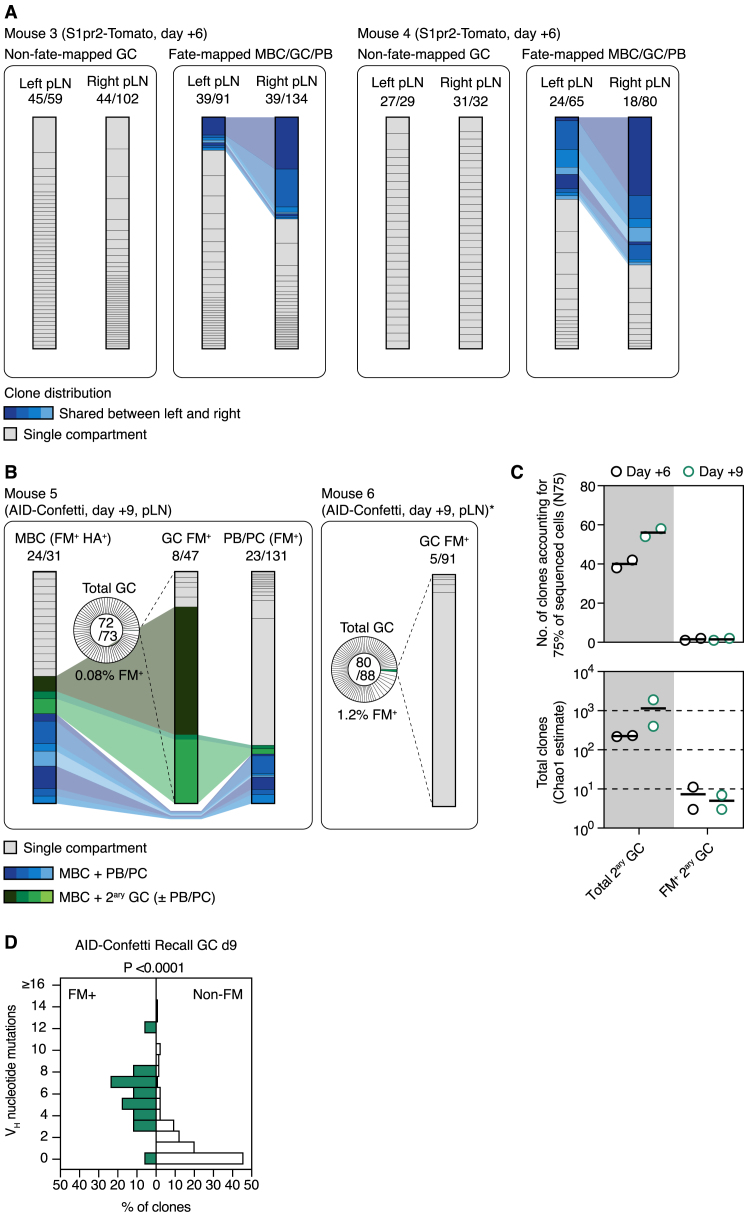
Comparative Clonal Composition of the Secondary Response to HA, Related to Figure 5 (A) Clonal sharing between footpads in fate-mapped and non-fate-mapped secondary GC B cells. S1pr2-Tomato mice were infected with influenza then boosted in both hind footpads with recombinant HA as described in Figure 5. Figures show the clonal composition of non-fate-mapped GC B cells (left panel) and fate-mapped MBC, PB, and GC B cells (right panel) in the two pLNs at 6 days after boost. Clones found in both left and right pLN are highlighted in blue. Data correspond to mice 3 and 4 from Figures 5A and S4A. (B) Clonal maps showing distribution of clones across different compartments. Data as in Figure 5C but analyzed at day 9 post boost. ^∗^MBCs and PBs for mouse 6 were not analyzed due to the very low number of cells. (C) Quantification of clonal diversity and dominance for the data in (B). (D) Average V_H_ mutations per clone in fate-mapped and non-fate-mapped GC B cells from (B). P value is for Mann-Whittney U test.

Secondary GCs were again highly diverse, with a median of >200 estimated total clones (Chao1) and N75 of 28 clones (Figure S4B). As with CGG immunization, these clones were largely unmutated at this stage (Figure S4C). Analysis of non-fate-mapped cells from S1pr2-Tomato mice revealed no clonal overlap between the left and right pLNs, again suggesting that this population derives primarily from precursors that were not previously clonally expanded (Figure S5A). In contrast, GC B cells from the fate-mapped minority were strikingly oligoclonal, with only 1–6 clones accounting for 75% of cells in all cases (Figures 5D and S4B). Results were essentially identical for the much larger GCs found at day 9 post-boost (Figures S5B–S5D). The secondary pLN PB compartment was also clonally restricted (median N75 = 5 and Chao1 = 25 clones at day 6 post-boost; Figure S4B), despite the large primary response induced by PR8 infection. Secondary GC and PB compartments were largely mutually exclusive (Figures 5C and S4A), in contrast to the marked overlap between compartments found for CGG immunization (Figure 4). Total clonality was higher in BM PCs (N75 = 22 and Chao1 = 129 clones), as expected given that this population is not restricted to clones induced by the HA boost. However, even though the precursors of BM PCs were fate-mapped during the primary response (predominantly directed to the influenza nucleoprotein; Allie et al., 2019), most expanded BM PC clones were inferred to be HA specific, given that they were also detected among pLN PBs and HA^+^ MBCs. Together with the observation that the titers of HA-specific IgG in serum rose by ∼10-fold upon boosting (Figure S3E), this suggests that a substantial fraction of the influenza-specific BM PCs must derive from the boosting of MBCs, rather than directly from the primary infection.

In contrast to recalled GC and PB and/or PC compartments, mLN and splenic MBCs were much more diverse, with an average of >200 and ∼100 estimated clones (Chao1), respectively. Plotting the clonal diversity (Chao1) and dominance (N75) of MBCs, secondary GCs and secondary PBs showed a clear segregation between memory and recalled compartments, indicative of a bottleneck restricting the access of MBC clones to the secondary response (Figure 5D). Memory clones could be divided roughly into two compartments: an oligoclonal compartment composed of clones also found among secondary GCs or PBs/PCs (“used” memory) and a highly diverse compartment comprising clones that were not detectably engaged by the secondary response (“unused” memory; Figures 5C and S4A). Used memory clones represented 38% of cells and 25% of clones in the splenic MBC compartment (with the caveat that the frequency of used MBCs could potentially be inflated by distal responses to boost antigen), but these same clones accounted for almost the entirety (83%) of secondary PBs (Figure S4D). Used and unused MBC clones differed in several other aspects: used clones tended to be more expanded (Figure 5E) and were more likely to be found across multiple MBC compartments (although unused memory was also on occasion found across compartments, indicating that at least part of this population is both clonally expanded and recirculating; Figure 5F). Used MBC clones were also found more frequently in the late primary mLN GC (Figure 5G), suggesting that they derived from clones that were successfully expanded and maintained in the primary response. Unused MBCs were more likely to contain IgM^+^ cells (Figure 5H) and had lower SHM loads (Figure 5I), implying that they originated predominantly from pre-GC or early GC stages. Total MBC clonal diversity in mLN, spleen, and pLN was estimated at ∼300 clones per mouse, of which a median of 27 were detectably recalled by boosting (Figure 5J).

We conclude that the relative inability of B cells with primary GC experience to reenter recall GCs and the clonal restriction of the PB compartment are common features of the secondary B cell response, regardless of the mode of priming. Such restriction occurs despite the presence of a clonally diverse but unused population of antigen-specific MBCs, indicating that boosting involves strong focusing on a broad MBC compartment onto a narrow subset of dominant clones.

### Secondary Responses Draw Repeatedly from a Limited Set of MBC Clones Characterized by Higher Germline Affinity

**Figure 6:**
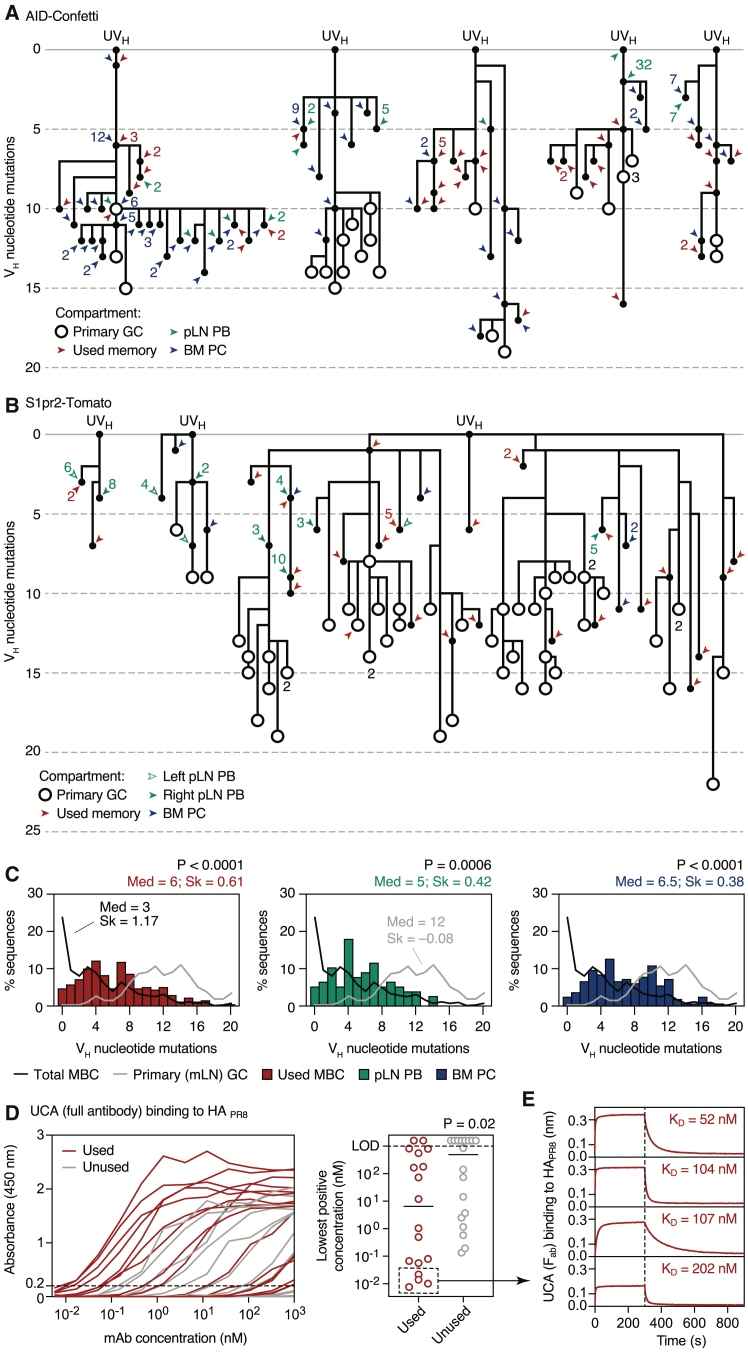
Repeated Recall of Dominant B Cell Clones into PB and PC Compartments (A and B) Phylogenetic trees showing relationship between V_H_ sequences (excluding CDR3) of cells from selected clones from Figures 5C and S4A, (A) AID-Confetti and (B) S1pr2-Tomato strains. UV_H_, unmutated V_H_ region. Open circles indicate cells present in primary (mLN) GC. Closed circles indicate sequences found in MBC, secondary (mLN) PB, or BM PC compartments (specified by the color of the adjacent arrowhead). Numbers indicate how many cells with a particular sequence were observed. (C) Histograms showing distribution of SHM in used MBCs, secondary pLN PBs, and BM PCs. Distributions for total MBCs and for the primary (mLN) GC are shown as lines for comparison. p values are for Mann-Whitney U test comparing the population of interest against total MBCs. Med, median; Sk, skewness (a measure of how skewed the distribution is toward the left [positive] or right [negative]). Data are pooled from two AID-Confetti and two S1pr2-Tomato mice (shown in Figures 5C and S4A) and collapsed by V_H_ sequence (cells with the exact same sequence are counted only once, to avoid skewing due to clonal expansion induced by the boost). (D) Binding to HA_PR8_ of monoclonal antibodies (mAbs) derived from the UCAs of used and unused MBCs. mAbs were cloned from 18 used and 18 unused MBCs and assayed for binding to recombinant HA_PR8_ by ELISA. (Left) graph shows mAb reactivity at 3-fold serial dilutions (each line represents one mAb). (Right) Lowest positive concentration (Absorbance at 450 nm >0.2) for each mAb. Each symbol represents one mAb. Data are representative of two experiments. p value is for Mann-Whitney U test. (E) Fab affinity for four used memory UCAs from (D) (dotted box), as measured by biolayer interferometry.

To gain insight into the histories of the MBC clones that dominate the secondary PB/PC response, we constructed mutational phylogenies for selected clones from the experiments described in Figures 5C and S4A and indicated the points from which MBCs and recall PCs were exported, as well as the position of any B cells still present in primary mLN GCs (Figures 6A and 6B). B cells from the primary mLN GC preferentially occupied more distal branches, as expected from their longer mutational history. Multiple SHM variants of each clone could usually be found among MBCs, indicative of repeated export throughout the history of the GC (Figures 6A and 6B). As with CGG priming (Figures 4F and 4G), local PBs could often be traced back to multiple independently recalled MBCs (in two more extreme cases, 6 distinct SHM variants of the same clone were detected among pLN PBs; Figures 6A and 6B). This effect was even more pronounced for BM PCs (21 SHM variants detected in the most extreme case; Figure 6A), although in this case direct recruitment from the primary (mLN) GC cannot be ruled out. Repeated recruitment of the same MBC clone was confirmed by the finding of different variants of the same clone populating the PB compartment in both right and left pLNs in S1pr2-Tomato mice in which both FPs had been boosted (Figures 6B and S5A).

MBCs were exported from points that spanned their clone’s entire phylogeny (Figures 6A and 6B, red arrowheads). Local PBs and BM PCs (green and blue arrowheads, respectively) were also scattered broadly, without consistent bias toward the recall of later, presumably more affinity matured, SHM variants. These trends were confirmed by global analysis of the SHM patterns of all recalled clones (Figure 6C). Whereas the distribution of SHM numbers among total MBCs (from both used and unused clones) was strongly skewed toward zero or few mutations, the distribution of used MBCs, pLN PBs, and BM PCs was evenly distributed across the SHM spectrum (as indicated by higher overall SHM and skewness levels that were closer to zero) and often included variants with zero V_H_ mutations. This implies that a clone’s initial V(D)J rearrangement, rather than time of export or extent of SHM and affinity maturation, is the dominant factor in determining MBC recall efficiency. To investigate this, we produced antibodies from the unmutated common ancestor (UCA) sequences of 18 used MBC clones from AID-Confetti mice (Figures 5C and S4A) and a matching number of unused MBC controls. Assaying the binding of these clones to H1_PR8_ by ELISA showed that, overall, used MBCs started from higher-affinity UCAs than unused MBCs (Figure 6D), implicating initial affinity as at least one of the factors that drives a naive B cell to generate recallable memory. Interestingly, several of the used memory UCAs clustered within a range that was beyond that of the highest binding unused memory UCA. Measurement of F_ab_ affinities for the top four UCAs in this cluster by biolayer interferometry (Figure 6E) showed that all four bound to HA_PR8_ with K_D_s in the 100 nM range (whereas unused F_ab_s had K_D_s that were too low to accurately measure), suggesting that germline affinities in this range favor the generation of recallable B cell memory.

We conclude that secondary PBs/PCs generated by homologous boosting derive from repeated recall of MBCs from a small subset of dominant clones generally derived from higher-affinity germline precursors, regardless of when these MBCs were exported into memory. This explains how a population of MBCs that is generated mostly in early (and clonally diverse) stages of the primary response can generate a secondary response that is clonally restricted and affinity matured. It also suggests that a clone’s germline affinity for antigen at the time of priming is a key factor in determining whether this clone can be efficiently recalled by homologous reimmunization.

## Discussion

Using realistic models of sequential antigen exposure at different anatomical sites, we reveal two prominent features of the clonal composition of the recall B cell response. First, rather than being composed primarily of previously matured B cells, secondary GC B cells were almost exclusively derived from a new repertoire of precursors without primary GC experience. This finding has implications for vaccination by sequential immunization, since it implies that this approach may benefit from strategies aimed at increasing the participation of previously mutated MBCs in secondary GCs. Second, only a minority of MBC-derived clones reenter GCs or contribute substantially to PB/PC compartments upon recall, while most of the diversity generated by priming is relegated to a pool of IgM^+^, low SHM MBCs not detectably engaged by the boost. This constriction of the MBC repertoire by boosting could potentially play a role in immunodominance, since decreased clonal diversity is likely to be associated with narrower epitope diversity.

The B cells that come to dominate secondary GCs are not labeled in either of our fate-mapping lines. Given the high efficiency of fate-mapping in both systems, this finding largely rules out prior GC experience among this dominant population. Moreover, parabiosis shows that naive B cells from the unimmunized parabiont are abundant in secondary GCs and, conversely, that the predominant precursors of secondary GCs are not in enough excess in the primed parabiont to skew secondary GC composition. These clones are also less likely to be shared between individual recall GCs in our photoactivation experiments (Figures 1C and 1D) or between right and left pLNs in S1pr2-Tomato mice (Figure S5A), again suggesting little if any prior clonal expansion. While these findings strongly suggest that most B cells in secondary GCs are naive derived, we cannot strictly rule out that these cells interacted with antigen in some form during primary immunization. We therefore refer to this population as “likely naive derived.” Regardless of the true nature of these cells, however, our findings suggest that the primary task of secondary GCs is to restart affinity maturation *de novo* rather than to refine previously matured MBCs. This preference may counteract the focusing of antibody responses on conserved epitopes and could explain why continuous exposure to influenza variants over life does not commonly lead to a robust cross-protective response to the conserved portions of HA (and may in fact actively move the response away from such epitopes (Ellebedy et al., 2014, Victora and Wilson, 2015, Wrammert et al., 2011)).

It should be emphasized that a limited number of MBC-derived and previously mutated clones were readily detectable in secondary GCs, and at least some of these clones underwent substantial expansion and rediversification upon boosting. Thus, productive participation of MBCs in secondary GCs is clearly possible, although relatively rare. These dynamics may explain the apparent discrepancy between our findings and those of prior studies (Dogan et al., 2009, McHeyzer-Williams et al., 2015, Pape et al., 2011, Zuccarino-Catania et al., 2014), which focused primarily on the populations of MBC-derived cells capable of reentering GCs rather than on the entirety of the secondary GC response. The ability of mutated MBCs to enter GCs with some efficiency provides an avenue that, if better understood, could be exploited for vaccination strategies that require sequential affinity maturation (Burton et al., 2012).

Although several hundred B cell clones can be engaged by primary protein immunization even in a single LN (Tas et al., 2016), recall GC and PB responses are consistently dominated by fewer than 10 MBC-derived clones. We found that at least part of this missing primary diversity resides within a highly diverse MBC compartment that is composed primarily of less expanded clones of predominantly IgM isotype and lower SHM content, suggesting these cells spent little if any time in a GC. Although the significance of this reservoir is unclear, these cells may represent a pool of antigen-experienced B cell clones that are pre-authorized to quickly become PCs upon restimulation with distantly related antigens. Evidence of this type of response can be found in human studies in which subjects mount rapid MBC-derived PB responses to antigens that they have not previously encountered, such as HA H5 and *Plasmodium falciparum* circumsporozoite protein (Ellebedy et al., 2014, Murugan et al., 2018).

By contrast, the minority of MBC clones engaged by homologous boosting was drawn from repeatedly, with no apparent bias toward recalling earlier or later SHM variants but with a preference for clones with higher germline affinity. This suggests that the properties of a clone’s initial V(D)J rearrangement, including its initial affinity for antigen, are a stronger predictor of whether this clone will be recalled than the extent of its affinity maturation or the overall timing of MBC export from the primary response. This model accommodates both the landmark finding by Weisel and Shlomchik (Weisel et al., 2016) that most MBCs derive from pre-GC or early GC phases (and must therefore be clonally diverse and low in SHM and affinity) and the classic hapten-carrier studies suggesting that secondary responses are not only higher in average affinity and SHM content than primary responses (Berek and Milstein, 1987) but also appear to be clonally restricted (Blier and Bothwell, 1987, Liu et al., 1996).

The reason for the paucity of MBC-derived B cells in recall GCs is unclear. Although current models of clonal selection and affinity maturation imply that MBCs should be both higher in affinity and present at higher precursor frequencies than naive B cells specific for the same antigen (Weisel and Shlomchik, 2017), the relatively inefficient generation of MBCs seen in experiment (Krishnamurty et al., 2016, Liu et al., 1996, Purtha et al., 2011, Weisel et al., 2010) suggests that the number of MBCs generated by a single antigen encounter is exceedingly small, especially when spread across subsets with different propensities to enter GC reactions (Dogan et al., 2009, Onodera et al., 2012, Pape et al., 2011, Zuccarino-Catania et al., 2014). Conversely, our previous work shows that the number of naive B cells with sufficient affinity to enter a primary GC reaction is quite large (Tas et al., 2016), likely in the several hundreds even for a single antigen. This number could be increased even further by the greater availability of T cell help in recall responses, which can dramatically lower the affinity required for naive B cells to access the GC (Schwickert et al., 2011, Silver et al., 2018). This “numbers game” could provide a simple explanation for why secondary GCs would be dominated by naive-derived B cells and would not require specific mechanisms suppressing MBC GC reentry. On the other hand, priming strategies that should in principle generate a much larger primary response, such as i.p. immunization or influenza infection, failed to flip the balance of secondary GCs toward MBCs or to increase the clonal diversity of recall PBs/PCs, suggesting that factors other than precursor numbers may also be at play. One such factor is negative feedback by antigen-specific antibody, which could limit GC entry of clones specific for epitopes targeted by serum antibody and has been found to affect both the selection of high-affinity cells in primary GCs (Zhang et al., 2013) and the magnitude of antigen-specific primary responses (Bergström et al., 2017). In adoptive transfer models, antibodies produced by IgG^+^ MBCs could inhibit the formation of secondary GCs by IgM^+^ MBCs, providing a precedent for such a model (Pape et al., 2011).

If applicable to humans, our findings have implications for vaccine design, since they imply that iteratively recruiting MBCs into recall GCs by repeated immunization is an inefficient process. Greater understanding of the mechanisms that govern MBC generation and propensity for recall should allow us to devise immunization approaches that allow greater GC reentry by MBCs, increasing the probability of sequential affinity maturation. Specific strategies may also be required to subvert the clonal focusing that occurs upon boosting, so as to favor less-dominant clones. These may include the use of immunogens specifically designed to escape antibody feedback by B cell clones already represented in serum (Dosenovic et al., 2015, Escolano et al., 2016), as well as avoiding the need for GC reentry entirely by refueling ongoing GCs in the same site (Dosenovic et al., 2015, Escolano et al., 2016, Shulman et al., 2013, Tian et al., 2016). Our data also suggest that, in order to be efficiently targeted by sequential immunization, an epitope must be rendered dominant from the very beginning. This provides theoretical support for efforts to target epitopes of interest by engineering high-affinity germline-targeting immunogens (Dosenovic et al., 2015, Jardine et al., 2016) or eliminating or occluding normally immunodominant regions (Bajic et al., 2019, Duan et al., 2018, Escolano et al., 2019, Kulp et al., 2017, Sun et al., 2019, Weidenbacher and Kim, 2019).

## STAR★Methods

### Key Resources Table

**Table:** 

REAGENT or RESOURCE	SOURCE	IDENTIFIER
**Antibodies**		

Anti-B220 BV785 (clone RA3-6B2)	BioLegend	RRID: AB_11218795
Anti-CD138 BV421 (clone 281-2)	BioLegend	RRID: AB_11204257
Anti-CD138 BV650 (clone 281-2)	BioLegend	RRID: AB_2650927
Anti-CD16/32 (Fc block) (clone 2.4G2)	Bio-X-Cell	RRID: AB_2687830
Anti-CD35 (clone 8C12) Alexa633	Tas et al., 2016	N/A
Anti-CD38 APC (clone 90/CD38)	BioLegend	RRID: AB_312933
Anti-CD38 PE/Cy7 (clone 90/CD38)	BioLegend	RRID: AB_2072892
Anti-CD4 PE (clone GK1.5)	BioLegend	RRID: AB_312692
Anti-CD4 V500 (clone RM4-5)	BD Biosciences	RRID: AB_1937315
Anti-CD45.2 AF700 (clone 104)	BioLegend	RRID: AB_493731
Anti-CD8a V500 (clone CD8a 53-6.7)	BD Biosciences	RRID: AB_1937317
Anti-CD95 PE/Cy7 (clone Jo2)	BD Biosciences	RRID: AB_396768
Anti-CXCR5 BV650 (clone L138D7)	BioLegend	RRID: AB_2562453
Anti-IgG1 FITC (clone RMG1-1)	BioLegend	RRID: AB_493293
Anti-IgG2a/b FITC (clone R2-40)	BD Biosciences	RRID: AB_394837
Anti-IgM APC-eFluor780 (clone II/41)	eBioscience	RRID: AB_2573983
Anti-PD1 APC/Cy7 (clone 29F.1A12)	BioLegend	RRID: AB_2563523
Anti-TCRb APC-eFluor780 (clone H57-597)	eBioscience	RRID: AB_1272173
Goat anti-human IgG-HRP	Southern Biotech	RRID: AB_2795644
Goat anti-mouse IgG-HRP	The Jackson laboratory	RRID: AB_2338506
Streptavidin BV421	BioLegend	Cat# 405231
Streptavidin BV650	BioLegend	Cat# 405226

**Bacterial and Virus Strains**		

Influenza A/Puerto Rico/08/1934 (mouse adapted PR8 strain), grown in embryonated chicken eggs	Carroll laboratory (Boston Children’s Hospital)	N/A

**Chemicals, Peptides, and Recombinant Proteins**		

TCL Buffer	QIAGEN	Cat# 1031576
ACK lysing buffer	Thermo Fisher Scientific	Cat# A10492-01
2-Mercaptoethanol	Thermo Fisher Scientific	Cat# 21985023
Tamoxifen	Sigma-Aldrich	Cat# T5648-5G
Corn oil	Sigma-Aldrich	Cat# C8267-500ML
Chicken gamma globulin	Rockland Immunochemicals	Cat# D602-0100
IgY	Gallus Immunotech	Cat# IgY-100
Imject Alum	Thermo Fisher Scientific	Cat# 77161
Addavax	Invivogen	Cat# vac-adx-10
Alhydrogel® adjuvant 2%	Invivogen	Cat# vac-alu-250
Meloxicam	Patterson Veterinary	Cat# 26637-621-0
Tween20	Sigma-Aldrich	Cat# P9416-50ML
Bovine Serum Albumin (BSA)	Sigma-Aldrich	Cat# A9647-500G
EDTA (0.5 M), pH 8.0, RNase-free	Thermo Fisher Scientific	Cat# AM9261
H1 A/Puerto Rico/8/1934 (Y98F)	A. McDermott (VRC/NIAID/NIH)	N/A
H1 A/Puerto Rico/8/1934 (Cysteine-stabilized)	This paper (Data S2)	N/A
H1 A/California/07/2009 (Cysteine-stabilized)	This paper (Data S2)	N/A
H5 A/Indonesia/05/2005 (Cysteine-stabilized)	This paper (Data S2)	N/A

**Critical Commercial Assays**		

Agencourt RNAClean XP kit	Beckman Coulter	Cat# A63987
CD43 MicroBeads	Miltenyi Biotec	Cat# 130-049-801
BirA-500 ligase kit	Avidity	Cat# BirA500
Zeba desalting column purification	Thermo Fisher Scientific	Cat# 89883
RT maxima reverse transcriptase	Thermo Fisher Scientific	Cat# EP0753
MACS Cell Separation Column LS	Miltenyi Biotec	Cat# 130-042-401
MiSeq Reagent Nano Kit v2 (500-cycles)	Illumina	Cat# MS-103-1003
CD43 (Ly-48) microbeads, mouse	Miltenyi Biotec	Cat# 130-049-801
Protein G Sepharose 4 Fast Flow	GE Healthcare	Cat# 17-0618-01
Ni Sepharose Excel	GE Healthcare	Cat# 17371201
High Precision Streptavidin (SAX) Biosensors	ForteBio	Cat# 18-5188
FreeStyle 293 Expression Medium	Thermo Fisher Scientific	Cat # 12338026
OptiPRO SFM medium	Thermo Fisher Scientific	Cat# 12309019
ProCHO5 medium	Lonza	Cat# 12-766Q

**Experimental Models: Cell Lines**		

Human: Freestyle 293F cells	Thermo Fisher Scientific	Cat# R79007
Chinese Hamster: CHO-DG44 cells	D. Hacker (EPFL)	N/A
	(Rajendra et al., 2017)	

**Experimental Models: Organisms/Strains**		

Mouse: C57BL6/J	The Jackson Laboratory	JAX: 000664
Mouse: PA-GFP	The Jackson Laboratory	JAX: 022486
Mouse: Ighg1^Cre^ (γ1-Cre)	The Jackson Laboratory	JAX: 010611
Mouse: *Aicda*^CreERT2^	C-A Reynaud, J-C Weill (U. Paris-Descartes)	N/A
	(Dogan et al., 2009)	
Mouse: Rosa26^Confetti/Confetti^	The Jackson Laboratory	JAX: 017492
Mouse: Rosa26^Stop-tdTomato^ (AI14)	The Jackson Laboratory	JAX: 007914
Mouse: *S1pr2*^CreERT2^ BAC transgenic	T. Kurosaki, T. Okada (RIKEN Yokohama)	N/A
	(Shinnakasu et al., 2016)	
Mouse: B6.SJL-Ptprc^a^ Pepc^b^/BoyJ	The Jackson Laboratory	JAX: 002014

**Oligonucleotides**		

See Data S3	IDT	N/A

**Recombinant DNA**		

Plasmid: pVRC8400	A. McDermott (VRC/NIAID/NIH)	N/A

**Software and Algorithms**		

Prism software	Graphpad	RRID: SCR_002798
FlowJo software	Treestar	RRID: SCR_008520
GC tree	DeWitt et al., 2018	N/A
Excel	Microsoft	RRID:SCR_016137
Illustrator	Adobe	RRID:SCR_010279
ImageJ	https://imagej.net/Welcome	RRID: SCR_003070
Photoshop	Adobe	RRID:SCR_014199
Gini index calculator	http://shlegeris.com/gini	N/A
EstimateS software	Colwell et al., 2012	N/A
IMGT database	Lefranc et al., 2009	http://www.imgt.org/
Vbase2 database	Retter et al., 2005	http://www.vbase2.org/
PandaSeq	Masella et al., 2012	N/A
FASTX toolkit	http://hannonlab.cshl.edu/fastx_toolkit	N/A
ForteBio Octet Analyzer Software Version 10	https://mdc.custhelp.com/app/answers/detail/a_id/20503/∼/octet-software-version-and-download-request	N/A

### Lead Contact and Materials Availability

Further information and requests for reagents should be directed to the Lead Contact Gabriel D. Victora, victora@rockefeller.edu. All unique reagents generated in this study are available with a completed Materials Transfer Agreement.

### Experimental Model and Subject Details

#### Mice

Mice were held in the Immunocore clean facility at the Rockefeller University under specific pathogen-free conditions. All mice were healthy, immune competent and drug and test naive prior to use in experiments. All mouse procedures were approved by the Rockefeller University’s Institutional Animal Care and Use Committee. Wild-type C57BL/6, *Ighg1*^Cre^ (also known as γ1-Cre) (Casola et al., 2006), *Rosa26*^Stop-tdTomato^ (AI14) (Madisen et al., 2010), and *Rosa26*^Confetti/Confetti^ (Snippert et al., 2010) mice were obtained from The Jackson Laboratory. PA-GFP mice were maintained at Rockefeller University (Victora et al., 2010). *S1pr2*^CreERT2^ BAC-transgenic mice (Shinnakasu et al., 2016) were generated by T. Kurosaki and T. Okada at RIKEN-Yokohama. *Aicda*^CreERT2^ mice (Dogan et al., 2009) were a kind gift from Jean-Claude Weill and Claude-Agnès Reynaud (Université Paris-Descartes). *Aicda*^CreERT2^ and *Rosa26*^Confetti^ strains were generated in 129/Ola embryonic stem cells and were backcrossed to the C57BL6 background for several generations prior to arrival to our laboratory. Trace genetic material from the 129/Ola strain still remains in this line, including the *Igh*^a^ allele, which is present in some individual mice (see “*Sequence analysis*” below for a description of how this is accounted for in the analysis of SHM patterns).

#### Cell lines

Cell lines were used for the production of recombinant antibodies and HA antigens. For antibody production, human Freestyle 293F cells were grown in FreeStyle 293 Expression Medium at 37°C, 8% CO2. Suspension-adapted Chinese Hamster Ovary (CHO)-DG44 cells (Rajendra et al., 2017) were grown in ProCHO5 medium at 37°C and 5% CO2. After transfection with HA-encoding plasmids, the temperature was lowered to 31°C.

### Method Details

#### Immunizations, infections, and treatments

GCs were induced in the right draining pLN of 7- to 12-week-old mice by s.c. immunization of the right FP with 10 μg of crude chicken gamma globulin (Rockland Immunochemicals) or purified IgY (Gallus Immunotech) interchangeably (we refer to both as CGG), supplemented with 1/3 volume of Imject Alum (ThermoScientific). After 30 days, the same formulation was administered to the left FP to generate an immune response in the left draining pLN. Alternatively, CGG was prepared with 1/2 volume of Addavax squalene-based antigen or 1/3 volume of aluminum hydroxide gel (alhydrogel) (both from Invivogen), as prescribed by the manufacturer. Recombination of the reporter alleles in S1pr2-Tomato and Aid-Confetti mice was induced during the early primary GC by administering respectively two or three doses of 12.5 mg tamoxifen (Sigma) dissolved in corn oil at 50 mg/ml, delivered via oral gavage between days 4 and 8 after immunization. Influenza infections were carried out intranasally with ∼33 PFU of mouse-adapted PR8 virus produced in embryonated chicken eggs (virus kindly provided by M. Carroll, Harvard University Medical School). Fate-mapping was carried out using the same dose of tamoxifen as for immunized mice, administered on days 7, 10, and 13 post-infection. For HA immunizations, trimer-stabilized HA (see below) was prepared with 1/3 volume of alhydrogel, as prescribed and 5 μg was administered in the appropriate FP.

#### Parabiosis

C57BL6 mice (CD45.2) mice were primed with CGG-alum either i.p. (50 μg) or s.c. in one FP (10 μg). After 4 weeks, immunized mice were parabiotically joined to naive congenic B6.SJL (CD45.1) mice. Parabiosis was performed in accordance to previously published protocols (Coleman and Hummel, 1969, Harris, 1997). Briefly, mice were placed under isoflurane anesthesia (1.5%–2%), and a longitudinal incision was made along one flank of each mouse, around 2 inches from the elbow to the knee. Mice were joined by the femurs and humeri by suturing, and skin was joined using sutures and wound clips. Meloxicam (2 mg/kg) was administered subcutaneously immediately prior to surgery and every 24 h after surgery for three days for analgesia. 3-4 weeks after surgery, the naive (CD45.1) parabiont was immunized with CGG-alum in the FP contralateral to the parabiont. The draining pLN was harvested and analyzed by flow cytometry 6 days after boost. Only mice with naive B cell chimerism > 25% were included in the analysis. CD45 alleles were reversed in some experiments to control for potential allele-specific effects.

#### Recombinant HA protein

Recombinant HA proteins used for immunization were produced in-house by transient transfection of suspension-adapted Chinese hamster ovary (CHO-DG44) cells (Rajendra et al., 2017). The coding sequences for H1 A/Puerto Rico/08/1934, (mouse adapted), H1 A/California/07/2009, and H5 A/Indonesia/05/2005, all truncated 5′ of the transmembrane domain (see Data S2 for sequences), were cloned into expression plasmid pVRC8400, which contains a C-terminal thrombin cleavage site followed by a foldon domain, an AviTag for biotinylation, and a His-tag for purification with Ni-Sepharose excel resin (GE Healthcare). To create trimer-stabilizing disulfide bonds, cysteine residues were introduced into residue positions L37 and G390 (H1_PR8_ numbering) as described for H1 A/Puerto Rico/8/1934 and H1 A/California/04/2009 (Lee et al., 2015). The equivalent residues were mutated for H5 A/Indonesia/05/2005 as shown in Data S2. HA used for immunization was treated with thrombin to remove domains not native to HA, and subsequently purified using a HiLoad 16/600 Superdex 200 prep-grade column (GE Healthcare) on an ÄKTA Purifier FPLC system (Amersham Pharmacia Biotech) prior to storage in PBS. HA tetramers for flow cytometry were generated by site-specific biotinylation of non-cysteine-stabilized treated HA protein containing the Y98F mutation that prevents sialic acid binding using BirA-500 ligase (Avidity), followed by Zeba desalting column purification (Thermo Fisher). Biotinylated HA was incubated with Streptavidin-BV650 in PBS for 30 min at RT at a molar ratio of 4 to 1 (HA-trimer to Streptavidin). The plasmid used for HA cloning and expression (pVRC8400) and proteins for tetramer construction were kindly provided by A. McDermott (VRC/NIAID/NIH).

#### Microscopy

LNs were harvested at different time points after primary or boost immunization, cleared of adipose tissue under a dissecting microscope, and placed in PBS between two coverslips held together by vacuum grease. Throughout the preparation and imaging, the tissue was kept on a cooled metal block. Multiphoton imaging was performed as described (Tas et al., 2016), using an Olympus FV1000 upright microscope fitted with a 25X 1.05NA Plan water-immersion objective and a Mai-Tai DeepSee Ti-Sapphire laser (Spectraphysics). To label FDC networks *in vivo*, a non-blocking antibody to CD35 (clone 8C12) conjugated to Alexa-633 was administered intravenously 24-48 hours prior to imaging. LN tissues isolated from AID-Confetti mice were imaged at λ = 930 nm for Confetti colors and at λ = 850 nm for Alexa 633. To image medullary regions, explanted LNs were embedded in low-melt agarose and cut into 300 μm slices using a Leica VT1000 S vibratome, as described (Tas et al., 2016). To isolate individual GCs for single-cell sorting, we performed photoactivation using PAGFP-transgenic mice crossed to γ1-cre and *Rosa26*^Stop-tdTomato^ as described (Tas et al., 2016). Briefly, clusters of tdTomato^+^ cells were identified by imaging at λ = 950 nm, at which no photoactivation is observed, and 3D regions of interest were photoactivated by higher-power scanning at λ = 830 nm.

#### Flow cytometry

For flow cytometry and cell sorting, cell suspensions of LNs were obtained by mechanical disassociated with disposable micropestles (Axygen). Spleens were homogenized by filtering through a 70-μm cell strainer and red-blood cells were lysed with ACK buffer (Thermo Scientific). Samples were enriched for B cells prior to flow cytometry and sorting by negative selection using anti-CD43-coupled magnetic beads (Miltenyi Biotec). BM cells were extracted by centrifugation of punctured tibiae and femurs at up to 10,000 x G for 10 s, then treated with ACK red blood cell lysing buffer. Cells from each tissue were resuspended in PBS supplemented with 0.5% BSA and 1mM EDTA and incubated for 30 min on ice with various fluorescently-labeled antibodies (see STAR Methods Key Resources Table). Cells were filtered and washed with the same buffer before analysis or sorting on BD FACS LSR II, FACS ARIA II, or FACS Symphony cytometers. Data were analyzed using FlowJo.

#### Single cell immunoglobulin sequencing

Single B cells were index-sorted into 96-well plates containing 5 μl TCL buffer (QIAGEN) supplemented with 1% β-mercaptoethanol. Nucleic acids were extracted using SPRI bead cleanup as described (Tas et al., 2016, Trombetta et al., 2014). RNA was reverse-transcribed into cDNA using RT maxima reverse transcriptase (Thermo Scientific) and oligo(dT) as a primer. Ig heavy chains were amplified by PCR using a forward primer with a consensus sequence for all V-region and reverse primers for each isotype. Ig kappa light chains were amplified separately where needed to confirm clonality or for antibody production purposes. Subsequently, 5-nucleotide barcodes were introduced by PCR to label Ig-sequences with plate- and well-specific barcodes. The forward primer contained barcodes to identify the plate and row number; the reverse primers contained the column-position barcode, adapted from (Han et al., 2014). In the final PCR step, Illumina paired-end sequencing adapters were incorporated into single-well amplicons. PCR-products were pooled by plate and cleaned-up using SPRI beads (0.7x volume ratio). Finally, the pooled amplicon library was sequenced with a 500-cycle Reagent Nano kit v2 on the Illumina Miseq platform as per the manufacturer’s instructions. Primer sequences are provided in Data S3.

#### Sequence analysis

Paired-end sequences were assembled with PandaSeq (Masella et al., 2012) and processed with the FASTX toolkit. The resulting demultiplexed and collapsed reads were assigned to wells according to barcodes. High-count sequences for every single cell/well were analyzed. Ig heavy chain and Ig light sequences were aligned to the online databases to determine the V(D)J arrangements and the number of somatic mutations compared to putative germline precursors. Given the presence of both IgM_a_ and IgM_b_ alleles in AID-Confetti mice, we aligned our sequences to both the IMGT (Lefranc et al., 2009) and Vbase2 (Retter et al., 2005) databases, choosing the assignment yielding the lowest number of somatic mutations in case of discrepancy. Sequences with a common V_H_/J_H_ gene and the same CDR3 length were grouped and classified automatically into clonal lineages if CDR3 nucleotide identity was 75% or higher. All sequences were then manually curated based on characteristics such as V-region SHM patterns and presence of stretches of mismatches at junctional regions. This resulted in further joining of sequences deemed to belong to the same clone, but which fell below 75% CDR3 nucleotide identity. Assignments were confirmed by light chain sequencing of all expanded clones from Figures 5C and S4A as well as selected clones from other experiments (see Data S1). For the minority of clones in which a rearranged *Igh* gene was not detected, clonality was established using *Igk* sequence. V_H_ mutation analyses were restricted to cells with productively rearranged *Igh* genes. Clonal lineage trees were inferred with GCtree (DeWitt et al., 2018), using the unmutated V gene sequence of the V(D)J clonal rearrangement for outgroup rooting.

#### Monoclonal antibody production and binding measurements

Heavy and light chain sequences were obtained from 18 used MBC clones found in AID-Confetti mice plus an equal number of clones from the unused MBC compartment. To avoid bias from random sampling, used MBC clones were selected by first ordering them by HA tetramer fluorescence intensity (measured by FACS) and picking every nth clone so that the 18 clones spanned the entire fluorescence range. For each used clone, we picked one unused control with matching HA fluorescence. Sequences were synthesized and assembled into Ig production vectors by Twist Biosciences. Plasmids were transfected into 293F cells and mAbs and Fabs (his-tagged) were purified using respectively protein-G or Ni-NTA affinity chromatography as described (Tas et al., 2016). Antibody binding was determined by ELISA as described (Tas et al., 2016). Briefly, wells were coated with 2 μg/ml recombinant HA (pre-thrombin treatment) in PBS at 4°C overnight. Wells were blocked with 2.5% BSA for 1-2 h at room temperature after 3 washes with PBS. mAbs were diluted to the specified concentrations in PBS supplemented with 0.5% BSA and 0.05% Tween20 and incubated in the wells for 1 h. Wells were washed 4 times with PBS with 0.05% Tween20 (PBS-T) before incubation with anti-human IgG conjugated to horse radish peroxidase (HRP). To determine serum titers of anti-HA_PR8_ IgG, ELISA was performed using 3-fold serial dilutions of serum samples starting at 1/100 and detection with anti-mouse IgG-HRP, followed by development with TMB (slow kinetic form, Sigma). Absorbance at 450 nm was measured on a Fisher Scientific accuSkan FC plate reader. Titers were determined as lowest concentration to reach an absorbance of 0.2 (interpolated linearly from the dilutions immediately above and immediately below 0.2 after background subtraction). Affinity measurements were carried out on a ForteBio Octet Red96 instrument as described (Tas et al., 2016). High Precision Streptavidin sensors were loaded with 5 μg/ml biotinylated HA_PR8_ and 600 nM Fabs in PBS supplemented with 0.1% BSA and 0.02% Tween20. Affinities were determined by partial fitting after subtraction of the HA-only background, using ForteBio Octet Analysis Software v. 10 software.

### Quantification and Statistical Analysis

The Gini index was used to determine the evenness of clonal distribution across a population, and was calculated using the online tool available at http://shlegeris.com/gini (last accessed 07/18/2019). The Chao1 index was used as an estimator of total clonal richness, as described (Chao, 1984, Tas et al., 2016). Calculations were performed using EstimateS software (Colwell et al., 2012). The Chao1 index provides a rough estimation of the lower bound of the total number of clones present in an ensemble, including any smaller clones. To estimate the diversity of the most expanded clones in numbers (rather than as a fraction, as with the more commonly used D50 and D75 indices), we calculated the number of clones accounting for 75% of sequenced cells, which we termed the “N75” index. This measurement is relatively insensitive to sampling depth if the proportion of singletons in the sample does not exceed 25%, but is sensitive to sampling when samples contain > 25% singletons. Therefore, this index will tend to underestimate the clonality of highly diverse samples such as MBCs. Statistical tests used to compare conditions are indicated in figure legends. Statistical analysis was carried out using GrahPad Prism v.8. Flow cytometry analysis was carried out using FlowJo v.10 software. Skewness was calculated in GraphPad Prism using the g1 method. Graphs were plotted using Prism v.8 and GCtree, and edited for appearance using Adobe Illustrator CS. Statistical details of experiments are provided in the results, figures and corresponding figure legends. Ig sequencing data is available in Data S1.

## Supplemental Information

Data S1. Ig Sequences, Related to Figures 1, 4, 5, and 6

Data S2. Amino Acid Sequences of Recombinant HAs, Related to Figures 2, 5, and 6

Data S3. Primers Used for Ig Sequencing, Related to Figures 1, 4, 5, and 6
